# The Study of Cancer Susceptibility Genes

**DOI:** 10.3390/cancers13092258

**Published:** 2021-05-08

**Authors:** Youri I. Pavlov

**Affiliations:** 1 Eppley Institute for Research in Cancer and Allied Diseases, University of Nebraska Medical Center, Omaha, NE 68198, USA; ypavlov@unmc.edu; 2 Departments of Biochemistry and Molecular Biology, Microbiology and Pathology, Genetics Cell Biology and Anatomy, University of Nebraska Medical Center, Omaha, NE 68198, USA; 3 Department of Genetics and Biotechnology, Saint-Petersburg State University, 199034 Saint-Petersburg, Russia

“…most complex, new direction for cancer medicine is to integrate our understanding of aberrant genes and pathways to explain the behavior of cancer as a whole, thereby renewing the cycle of knowledge, discovery and therapeutic intervention.”—“The Emperor of all Maladies” [1].

Cancer is a disease caused by uncontrolled cell growth driven by genetic changes and the misregulation of genetic information expression. Cancer cells recklessly and selfishly propagate, neglecting the organized developmental scenarios for multicellular organisms, harming the host and eventually themselves. Faults in cell growth control are caused by a plethora of causes: point mutations, copy number variations, chromosome rearrangements, epigenetic alterations, or aberrant forms of essential regulator proteins in the form of amyloids. In few cases, there is a single primary initiation event, but in most cases, a cascade of events causes changes in regulatory networks that result in tumor growth and invasiveness. Defects in the maintenance and expression of hereditary information predispose cells to cancer and influence the outcomes of therapeutic interventions. The current Special Issue of Cancers collects five reviews and 13 experimental papers devoted to the factors playing roles in predisposition to cancer and, in clear documented cases, hereditary forms of cancer. These papers represent a collaborative, international effort revealing the complexity of the problem.

The innovative parallel analysis of cancer-specific DNA sequence variations in genomes and gene expression, affecting not individual genes but molecular pathways falling into four functional groups: signaling, metabolic, cytoskeleton, and DNA repair, revealed that the latter group’s genes had the highest mutation enrichment, and upregulation levels [2]. The signaling and cytoskeleton group members were enriched by the genes with many SNVs and experienced the most substantial downregulation of gene expression, alluding to their possible roles as initiators in carcinogenesis. The deregulation of gene expression in angiosarcoma in a *TP53*-rat model revealed equal numbers of upregulated and downregulated genes [3]. Consistent with the previous study, upregulated genes belong to the DNA repair group and include genes for DNA helicases, chromosome maintenance complexes, recombination, and replication. The Functional Signature Ontology (FUSION) approach of genome-wide, loss-of-function screening is another way to identify critical genes in cancer and find vulnerabilities of tumor cells [4]. A gene expression-based high-throughput screening method allows researchers to identify novel therapeutic targets.

Recent studies on tumor genomes revealed that mutations in replicative DNA polymerase genes caused a predisposition for cancer by increasing genome instability. However, out of three DNA polymerases operating at the replication fork, colon and endometrial cancer-associated mutations (both sporadic and hereditary) predominantly affect the catalytic subunit of polymerase ε that participates in leading strand DNA synthesis [5,6], suggesting a unique role that this polymerase plays during replication and human development. It is well established that defects in DNA mismatch repair (Lynch syndrome) predispose to colon and other cancers. The new study found that the condition can also lie in the etiology of sarcomas [7]. Fanconi anemia is connected to the defects of machinery dealing with the aberrant replication of damaged DNA with inter-strand DNA crosslinks. The review [8] highlights the importance of Fanconi anemia genes in noncanonical pathways, such as mitochondria homeostasis, inflammation, and virophagy. The primary defects in DNA repair mechanisms in such patients could be exacerbated by an impairment of the cytoprotective pathways, contributing to the disease’s multifaceted manifestations.

The hereditary ovarian cancer and, commonly, breast cancer start early and in multiple generations. DNA methylation patterns provide a paradigm for the epigenetic inheritance of cancer susceptibility, with emphasis on co-methylated networks [9]. Multiple paragangliomas and papillary thyroid carcinoma are likely connected to mutations in the *DNMT3a* gene, responsible for de novo methylation [10]

Changes in developmental programs do not necessarily involve DNA or RNA. The widespread Alzheimer’s disease is connected to the accumulation of misfolded polymerized proteins, amyloid β (Aβ) and tau, in brains, and to neuronal degeneration, dementia, and death. Alzheimer’s disease patients have a lower incidence of cancer. The study published in this Special Issue revealed that the effect of cancer on the risk of death of Alzheimer’s depended on age and the time since cancer diagnosis [11]. Cancer-directed chemotherapy protects against Alzheimer’s disease-related death in patients diagnosed with breast cancer. Emerging evidence connects some forms of cancer to the accumulation of critical proteins (e.g., P53) in an amyloid form; therefore, these drugs may affect amyloids.

Several reports documented the roles of defined genes on particular cancer types: *CDH1* and *CTNNA1* encoding for proteins of the cadherin and catenin families, respectively, and thus cell adhesion, in hereditary diffuse gastric cancer and lobular breast cancer [12]; *TNFSF13* (April) and *TNSF13B* (TACI), genes for ligands of the tumor necrosis factor family in chronic lymphocytic leukemia [13]; and *SPON2* (Spondin 2), involved in O-linked glycosylation and ERK signaling in gastric cancer [14]. High levels of CES1 carboxylesterase, responsible for the hydrolysis or transesterification of various xenobiotics’ expression, predicted better outcomes for prostate cancer patients [15]. A polymorphism rs6942067 leading to a high transmembrane protein DCBLD1 expression worsens prognosis in human papillomavirus-negative head and neck squamous cell carcinoma [16]. Tumor susceptibility gene 101 (TSG101), encoding for an inactive homolog of ubiquitin-conjugating enzyme, plays a role in normal development and disease [17]. The interaction between two polymorphisms affecting PD-1 and PD-L1 immune checkpoints is connected to renal cancer risk [18]. 

Two contributions are in the area of genetic counseling. In patients with gastric and pancreatic adenocarcinoma, hereditary syndromes were associated with mutations in mismatch repair, *ATM*, *TP53*, *CDH1* (cadherin), and *BRCA* genes [19]. Another study provides evidence for the notion that genetic testing should be done with a broader group of women diagnosed with breast cancer than currently accepted [20].

In summary, this Special Issue of *Cancers* presents recent progress in the tantalizing task of finding factors contributing to cancer development. When the culprit is identified for a particular cancer type, life-saving strategies of treatment and support may become available, as has happened with specific cancer types, for example, chronic myelogenous leukemia, where treatment with tyrosine kinase inhibitors dramatically improved quality of life and survival. For other types of cancers, progress is being made, but still, there is a long road ahead in the battle of humanity with cancer, given the complexity of the disease and the multiplicity of causing factors. 
